
JOURNAL INFORMATION
==============================
NLM Title Abbreviation: ScientificWorldJournal
Journal ID: ScientificWorldJournal
NLM Journal ID: 101131163
Journal ID: TSWJ

The Scientific World Journal

ISSN: 2356-6140
EISSN: 1537-744X
Publisher: Wiley

ARTICLE INFORMATION
==============================
PMCID: PMC5848679
PMID: 18836656
DOI: 10.1100/tsw.2008.115
Article ID: 284341
Article version: 1
Subjects: Directions in Science

The microRNA-200 Family Regulates Epithelial to Mesenchymal Transition

Paterson Emily L. 1 *
Kolesnikoff Natasha 1
Gregory Philip A. 1 2
Bert Andrew G. 1
Khew-Goodall Yeesim 1 3
Goodall Gregory J. 1 2
1 Hanson Institute and Division of Human Immunology, Institute of Medical and Veterinary Science, Adelaide, SA 5000, Australia
2 Discipline of Medicine, The University of Adelaide, Adelaide, SA 5005, Australia
3 School of Molecular and Biomedical Sciences, The University of Adelaide, Adelaide, SA 5005, Australia
*Emily L. Paterson: emily.paterson@imvs.sa.gov.auAcademic Editor: Adele Murrell

Publication date: 2008
Electronic publication date: 2008 Sep 21
Volume: 8
First page: 901
Last page: 904
Received 2008 Aug 1; Revised 2008 Aug 14; Accepted 2008 Aug 14
Copyright: Copyright © 2008 Emily L. Paterson et al.
Copyright year: 2008
License: This is an open access article distributed under the Creative Commons Attribution License, which permits unrestricted use, distribution, and reproduction in any medium, provided the original work is properly cited.
License URL: https://creativecommons.org/licenses/by/3.0/

Keywords: miR-200, epithelial to mesenchymal transition, ZEB1, SIP1

==============================
Directions in Science
*TheScientificWorldJOURNAL* (2008) 8, 901–904
ISSN 1537-744X; DOI 10.1100/tsw.2008.115

TheScientificWorldJOURNAL
www.thescientificworld.com

### The microRNA-200 Family Regulates Epithelial to Mesenchymal Transition

Emily L. Paterson[1,*], Natasha Kolesnikoff[1,*], Philip A. Gregory[1,2], Andrew G. Bert[1], Yeesim Khew-Goodall[1,3], and Gregory J. Goodall[1,2,**]

*[1]Hanson Institute and Division of Human Immunology, Institute of Medical and Veterinary Science, Adelaide, SA 5000, Australia; [2]Discipline of Medicine, The University of Adelaide, Adelaide, SA 5005, Australia; [3]School of Molecular and Biomedical Sciences, The University of Adelaide, Adelaide, SA 5005, Australia*

**E-mail:** emily.paterson@imvs.sa.gov.au, natasha.kolesnikoff@imvs.sa.gov.au, philip.gregory@imvs.sa.gov.au, andrew.bert@imvs.sa.gov.au, yeesim.khew-goodall@imvs.sa.gov.au, greg.goodall@imvs.sa.gov.au

*Received August 1, 2008; Revised August 14, 2008; Accepted August 14, 2008; Published September 21, 2008*

**KEYWORDS:** miR-200, epithelial to mesenchymal transition, ZEB1, SIP1

The majority of human cancers originate from epithelial cells[1], with their local invasion and metastasis accounting for 90% of cancer-related death[2]. The progression of metastasis is a complex event thought to incorporate the reversible developmental process of epithelial to mesenchymal transition (EMT). This involves cancerous epithelial cells transitioning into motile mesenchymal cells, which invade distant sites in the body where they lodge and undergo mesenchymal to epithelial transition (MET) before proliferating into secondary tumours[3]. Essential to the maintenance of the polarised epithelial monolayer is the adherens junction protein, E-cadherin, which has been described as a tumour suppressor because its down-regulation is associated with invasion and metastasis[4]. During EMT, the increase of transcription factors, such as ZEB1, SIP1, Snail, and Slug, strongly represses the transcription of the E-cadherin gene, facilitating loss of the strong cell-cell interactions characteristic of epithelial cells.

First discovered in 1993, microRNAs (miRNAs) are an abundant class of noncoding, 18–25 nt, single-stranded oligoribonucleotides that function post-transcriptionally to negatively regulate the translation of messenger RNA (mRNA). Target recognition is based on complementary binding to the 3' untranslated region (3'UTR) of the target mRNA. Expression of miRNAs can vary from ubiquitous to highly site and/or temporal specific. They are predicted to regulate up to 30% of genes in eukaryotes[5] and have regulatory roles in cellular processes, such as proliferation[6,7,8], differentiation[9], apoptosis[10,11], metabolism[12], embryogenesis and developmental timing[13,14,15,16]. Consistent with their roles in maintaining normal cell function, the aberrant expression of miRNAs has been linked to oncogenesis[17] and, more recently, metastasis[18,19,20,21,22]. Since EMT is an essential developmental process and is implicated in metastasis, we postulated that miRNAs may be involved in its regulation.

Evidence that EMT is required for cancer metastasis is increasing with investigations into the signalling mechanisms driving EMT. A potent inducer of EMT is the cytokine transforming growth factor-β (TGF-β), which has been implicated in regulating transcription factors including Snail, Slug, ZEB1, SIP1, and basic-helix-loop-helix (bHLH) factors, such as Twist[23,24]. However, knowledge of the role of miRNAs and their potential target genes in EMT is limited. Previous studies have shown that

*These authors contributed equally to this manuscript.
**Corresponding author.

©2008 with author.
Published by TheScientificWorld; www.thescientificworld.com miR-200c and miR-200b post-transcriptionally repress ZEB1 and SIP1, respectively[25,26]. The differential expression of miRNAs in primary tumours and their metastases has been reported, but it remains to be elucidated as to whether miRNAs are involved in the initiation of invasion in the context of EMT.

We conducted microarray analysis to assess differential expression of miRNAs in an EMT system in Madin-Darby canine kidney (MDCK) cells, induced by the protein tyrosine phosphatase (PTP) Pez[27]. Overexpression of PTP-Pez in MDCK cells leads to induction of TGF-β signalling and EMT, as observed in cells losing cell-cell adhesion and adopting a mesenchymal phenotype[27]. Quantitative real-time PCR of miRNA expression in the MDCK-Pez model confirmed that all five members of the highly related miR-200 family (miR-200a, -200b, -200c, -141, and -429) and miR-205 were down-regulated over 100-fold following EMT[28]. In addition, a TGF-β–induced EMT model also showed similar differential expression of these miRNAs. Stable overexpression of the miR-200s prevented TGF-β–induced EMT, implicating the miR-200s as key regulators of EMT. Computational searches using TargetScan[29] revealed multiple potential binding sites for the miR-200s and miR-205 in the 3'UTRs of the mesenchymal markers, ZEB1 and SIP1. Transfection of luciferase reporters containing either the ZEB1 or SIP1 3'UTR into the Pez-induced EMT model showed high expression in mesenchymal cells, but reduced expression in epithelial cells, consistent with the differences in miR-200s and miR-205 levels in these cells. This was verified by cotransfection of the reporters with either antisense inhibitors or synthetic precursors to miR-200s and miR-205 in epithelial and mesenchymal cells, respectively. Inhibition of the miR-200s in MDCK cells over a 19-day time course was sufficient to induce EMT, as demonstrated by loss of E-cadherin, gain of ZEB1 and SIP1, and an increase in cell migration. ZEB1 and SIP1 were shown to be essential for EMT by siRNA-mediated knockdown of their levels in the presence of the miR-200 inhibitors, which prevented this transition. Conversely, ectopic expression of the miR-200s in mesenchymal cells induced MET. This led to the conclusion that the miR-200 family is able to regulate EMT by repressing the translation of ZEB1 and SIP1. We extended this study to include analysis of miRNA, E-cadherin, ZEB1, and SIP1 in a range of epithelial and mesenchymal breast cancer lines. Strikingly, the epithelial lines expressed miR-200s and E-cadherin, and lacked ZEB1 and SIP1, whereas the mesenchymal lines were deficient in miR-200s and E-cadherin, but had high levels of ZEB1 and SIP1. *In vivo* analysis of miR-200 expression was conducted via real-time PCR of primary human ductal and metaplastic breast cancers, which exhibit epithelial and mesenchymal cell morphology, respectively. The ductal tumours had high levels of E-cadherin and miR-200s, while the invasive mesenchymal metaplastic tumours lacked these markers. This indicates that loss of the miR-200s may result in a more aggressive cancer, leading to metastasis.

In summary, we demonstrated that the miR-200 family and miR-205 maintain the epithelial phenotype by repressing the translation of ZEB1 and SIP1. The importance of this finding is supported by a number of subsequent reports that also demonstrate this relationship of the miR-200 family with ZEB1 and SIP1[30,31,32]. Loss of the miR-200s in invasive metaplastic breast cancers implicates these miRNAs as potential therapeutic agents that may have the ability to confine carcinoma cells to the primary tumour site, where the mass can be effectively resected.

### REFERENCES

1. Christofori, G. (2006) New signals from the invasive front. *Nature* **441,** 444–450.
2. Sporn, M.B. (1996) The war on cancer. *Lancet* **347,** 1377–1381.
3. Huber, M.A., Kraut, N., and Beug, H. (2005) Molecular requirements for epithelial-mesenchymal transition during tumor progression. *Curr. Opin. Cell Biol.* **17,** 548–558.
4. Vleminckx, K., Vakaet, L., Mareel, M., Fiers, W., and Vanroy, F. (1991) Genetic manipulation of E-cadherin expression by epithelial tumor-cells reveals an invasion suppressor role. *Cell* **66,** 107–119.
5. Yu, Z.B., Jian, Z.F., Shen, S.H., Purisima, E., and Wang, E. (2007) Global analysis of microRNA target gene expression reveals that miRNA targets are lower expressed in mature mouse and Drosophila tissues than in the embryos. *Nucleic Acids Res.* **35,** 152–164.
6. Zhao, Y., Samal, E., and Srivastava, D. (2005) Serum response factor regulates a muscle-specific microRNA that targets Hand2 during cardiogenesis. *Nature* **436,** 214–220.

7. Cheng, A.M., Byrom, M.W., Shelton, J., and Ford, L.P. (2005) Antisense inhibition of human miRNAs and indications for an involvement of miRNA in cell growth and apoptosis. *Nucleic Acids Res.* **33,** 1290–1297.
8. Lee, Y.S., Kim, H.K., Chung, S.M., Kim, K.S., and Dutta, A. (2005) Depletion of human micro-RNA miR-125b reveals that it is critical for the proliferation of differentiated cells but not for the downregulation of putative targets during differentiation. *J. Biol. Chem.* **280,** 16635–16641.
9. Chen, C.Z., Li, L., Lodish, H.F., and Bartel, D.P. (2004) MicroRNAs modulate hematopoietic lineage differentiation. *Science* **303,** 83–86.
10. Chan, J.A., Krichevsky, A.M., and Kosik, K.S. (2005) MicroRNA-21 is an antiapoptotic factor in human glioblastoma cells. *Cancer Res.* **65,** 6029–6033.
11. Cimmino, A., Calin, G.A., Fabbri, M., Iorio, M.V., Ferracin, M., Shimizu, M., Wojcik, S.E., Aqeilan, R.I., Zupo, S., Dono, M., Rassenti, L., Alder, H., Volinia, S., Liu, C.G., Kipps, T.J., Negrini, M., and Croce, C.M. (2005) miR-15 and miR-16 induce apoptosis by targeting BCL2. *Proc. Natl. Acad. Sci. U. S. A.* **102,** 13944–13949.
12. Xu, P.Z., Vernooy, S.Y., Guo, M., and Hay, B.A. (2003) The Drosophila microRNA mir-14 suppresses cell death and is required for normal fat metabolism. *Curr. Biol.* **13,** 790–795.
13. Lee, R.C., Feinbaum, R.L., and Ambros, V. (1993) The C. elegans heterochronic gene lin-4 encodes small RNAs with antisense complementarity to lin-14. *Cell* **75,** 843–854.
14. Johnston, R.J., Chang, S., Etchberger, J.F., Ortiz, C.O., and Hobert, O. (2005) MicroRNAs acting in a double-negative feedback loop to control a neuronal cell fate decision. *Proc. Natl. Acad. Sci. U. S. A.* **102,** 12449–12454.
15. Johnston, R.J. and Hobert, O. (2003) A microRNA controlling left/right neuronal asymmetry in Caenorhabditis elegans. *Nature* **426,** 845–849.
16. Wienholds, E., Koudijs, M.J., van Eeden, F.J.M., Cuppen, E., and Plasterk, R.H.A. (2003) The microRNA-producing enzyme Dicer1 is essential for zebrafish development. *Nat. Genet.* **35,** 217–218.
17. Calin, G.A. and Croce, C.M. (2006) MicroRNA signatures in human cancers. *Nat. Rev. Cancer* **6,** 857–866.
18. Tavazoie, S.F., Alarcon, C., Oskarsson, T., Padua, D., Wang, Q.Q., Bos, P.D., Gerald, W.L., and Massague, J. (2008) Endogenous human microRNAs that suppress breast cancer metastasis. *Nature* **451,** 147–152.
19. Huang, Q.H., Gumireddy, K., Schrier, M., le Sage, C., Nagel, R., Nair, S., Egan, D.A., Li, A.P., Huang, G.H., Klein-Szanto, A.J., Gimotty, P.A., Katsaros, D., Coukos, G., Zhang, L., Pure, E., and Agami, R. (2008) The microRNAs miR-373 and miR-520c promote tumour invasion and metastasis. *Nat. Cell Biol.* **10,** 202–210.
20. Asangani, I.A., Rasheed, S.A.K., Nikolova, D.A., Leupold, J.H., Colburn, N.H., Post, S., and Allgayer, H. (2008) MicroRNA-21 (miR-21) post-transcriptionally downregulates tumor suppressor Pdcd4 and stimulates invasion, intravasation and metastasis in colorectal cancer. *Oncogene* **27,** 2128–2136.
21. Ma, L., Teruya-Feldstein, J., and Weinberg, R.A. (2007) Tumour invasion and metastasis initiated by microRNA 10b in breast cancer. *Nature* **449,** 682–688.
22. Sengupta, S., den Boon, J.A., Chen, I.H., Newton, M.A., Stanhope, S.A., Cheng, Y.J., Chen, C.J., Hildesheim, A., Sugden, B., and Ahlquist, P. (2008) MicroRNA 29c is down-regulated in nasopharyngeal carcinomas, up-regulating mRNAs encoding extracellular matrix proteins. *Proc. Natl. Acad. Sci. U. S. A.* **105,** 5874–5878.
23. Peinado, H., Quintanilla, M., and Cano, A. (2003) Transforming growth factor beta-1 induces snail transcription factor in epithelial cell lines - mechanisms for epithelial mesenchymal transitions. *J. Biol. Chem.* **278,** 21113–21123.
24. Peinado, H., Olmeda, D., and Cano, A. (2007) Snail, ZEB and bHLH factors in tumour progression: an alliance against the epithelial phenotype? *Nat. Rev. Cancer* **7,** 415–428.
25. Hurteau, G.J., Carlson, J.A., Spivack, S.D., and Brock, G.J. (2007) Overexpression of the microRNA hsa-miR-200c leads to reduced expression of transcription factor 8 and increased expression of E-cadherin. *Cancer Res.* **67,** 7972–7976.
26. Christoffersen, N.R., Silahtaroglu, A., Orom, U.A., Kauppinen, S., and Lund, A.H. (2007) miR-200b mediates post-transcriptional repression of ZFHX1B. *RNA* **13,** 1172–1178.
27. Wyatt, L., Wadham, C., Crocker, L.A., Lardelli, M., and Khew-Goodall, Y. (2007) The protein tyrosine phosphatase Pez regulates TGF beta, epithelial-mesenchymal transition, and organ development. *J. Cell Biol.* **178,** 1223–1235.
28. Gregory, P.A., Bert, A.G., Paterson, E.L., Barry, S.C., Tsykin, A., Farshid, G., Vadas, M.A., Khew-Goodall, Y., and Goodall, G.J. (2008) The mir-200 family and mir-205 regulate epithelial to mesenchymal transition by targeting ZEB1 and SIP1. *Nat. Cell Biol.* **10,** 593–601.
29. Lewis, B.P., Burge, C.B., and Bartel, D.P. (2005) Conserved seed pairing, often flanked by adenosines, indicates that thousands of human genes are microRNA targets. *Cell* **120,** 15–20.
30. Park, S.M., Gaur, A.B., Lengyel, E., and Peter, M.E. (2008) The miR-200 family determines the epithelial phenotype of cancer cells by targeting the E-cadherin repressors ZEB1 and ZEB2. *Genes Dev.* **22,** 894–907.
31. Korpal, M., Lee, E.S., Hu, G.H., and Kang, Y.B. (2008) The miR-200 family inhibits epithelial-mesenchymal transition and cancer cell migration by direct targeting of E-cadherin transcriptional repressors ZEB1 and ZEB2. *J. Biol. Chem.* **283,** 14910–14914.
32. Burk, U., Schubert, J., Wellner, U., Schmalhofer, O., Vincan, E., Spaderna, S., and Brabletz, T. (2008) A reciprocal repression between ZEB1 and members of the miR-200 family promotes EMT and invasion in cancer cells. *EMBO Rep.* **9,** 582–589.

**This article should be cited as follows:**

Paterson, E.L., Kolesnikoff, N., Gregory, P.A., Bert, A.G., Khew-Goodall, Y., and Goodall, G.J. (2008) The microRNA-200 family regulates epithelial to mesenchymal transition. *TheScientificWorldJOURNAL* **8**, 901–904. DOI 10.1100/tsw.2008.115.
